# Bone health assessment in adults with fragility fracture risk factors between 2002–2014: a retrospective cohort study

**DOI:** 10.3399/BJGPO.2023.0084

**Published:** 2024-01-24

**Authors:** Anup Bahadur Pradhan, Elaine Nicholls, John James Edwards, Victoria Welsh, Zoe Paskins

**Affiliations:** 1 School of Medicine, David Weatherall Building, Keele University, Keele, UK; 2 Belvidere Medical Practice, Shrewsbury, UK; 3 Keele Clinical Trials Unit, Keele University, Keele, UK; 4 Wolstanton Medical Centre, Wolstanton, UK; 5 Haywood Academic Rheumatology Centre, Midlands Partnership University NHS Foundation Trust, Haywood Hospital, Burslem, UK

**Keywords:** bone health assessment, fragility fracture, osteoporosis, men, primary health care

## Abstract

**Background:**

Lifetime risk of fragility fractures is 50% in post-menopausal women and 20% in men aged >50 years. Identifying people at high risk facilitates early intervention and reduction of biopsychosocial morbidity associated with these fractures.

**Aim:**

To explore if bone health assessment (BHA) rates differ between women and men aged ≥50 years with fragility fracture risk factors.

**Design & setting:**

A primary care-based cohort study in North Staffordshire, UK.

**Method:**

Patients were identified from the Consultations in Primary Care Archive (CiPCA) database between 2002 and 2014 with one or more fragility fracture risk factors (previous fractures, falls, and prolonged steroid use). Evaluation of BHA within 12 months of presentation of the first risk factor was carried out by searching for codes for fracture risk assessment tools (FRAX and QFracture), bone density measurement, specialist service referral, or if bone-protection medication was started.

**Results:**

A total of 15 581 patients with risk factors were identified; men represented 40.4% of the cohort. The study found 1172 (7.5%) had BHA performed within 1 year of presentation, and 8.9% of women and 5.5% of men had BHAs, which was found with strong statistical evidence (χ^2^ = 59.88, *P* = 1 × 10^–14^). This relationship prevailed after adjusting for other covariates, such as comorbidity and number of consultations, with an odds ratio of 1.25 (95% confidence interval [CI] = 1.08 to 1.43).

**Conclusion:**

This study has shown that rates of BHA were generally low and even lower in men compared with women. Primary care clinicians should be alert to fragility fracture risk factors in both men and women to enable early assessment and intervention.

## How this fits in

Half of all post-menopausal women and one-fifth of men aged >50 years are expected to sustain a fragility fracture during their lifetime. Identifying high-risk patients, to enable early intervention, may lower the number of fragility fractures. This study has shown that rates of bone health assessment (BHA) were low in both men and women with fragility fracture risk factors. Men had disproportionately lower assessment rates than women, representing an important inequality in management. It is recommended that primary care clinicians consider BHA in both men and women with fragility fracture risk factors.

## Introduction

Osteoporosis is a common metabolic condition characterised by low bone density with deterioration of the skeletal microarchitecture. This condition, referred to as the ‘silent disease’,^1^ affects 3.5 million people in the UK^2^ and is usually detected after fragility fractures are sustained.^3^ Common fracture sites include the hip, spine, forearm, and humerus; more than 500 000 fragility fractures occur annually in the UK,^4^ of which 76 000 are hip fractures.^5^ Fragility fractures are associated with poor physical, social, and psychological impacts including chronic pain and depression, while hip fractures increase mortality risk within 1 year by up to 20%.^6^

Although osteoporosis preferentially affects post-menopausal women, with fragility fractures seen in 50% of women, 20% of men aged >50 years are also expected to sustain a fragility fracture during their lifetime,^4^ with men carrying a higher mortality rate in the first year following hip fractures compared with women.^7^ Identifying men and women with fragility fracture risk factors and conducting an early BHA may prevent potentially life-threatening fractures from occurring, as evidence-based treatment to lower fracture risk is available.^8^

BHA includes a fracture risk assessment, measurement of bone density and/or starting bone-sparing medication. Early BHA is included in the national guidelines for the identification and management of osteoporosis, which apply to both men and women. Guidance includes a recommendation to calculate fracture risk in adults with risk factors including previous fragility fractures, use of oral corticosteroids, or a history of falls. Absolute fracture risk can be calculated by validated tools including FRAX or QFracture, with advice to perform bone density scanning, or treat with bone-sparing medication where appropriate.^3^

A previous study has shown a reduced rate of fracture risk calculation in populations of men compared with women,^9^ although this has yet to be examined in UK primary care. This study examined rates of BHA in men and women by using routinely collected electronic health record data from UK primary care to test the hypothesis that there is no difference in BHA rates between men and women with risk factors for fragility fractures.

## Method

A retrospective cohort study over a 12-year period (2002–2014) was conducted using routinely collected electronic primary healthcare data from the Consultations in Primary Care Archive (CiPCA) database, which is a resource linking individual patient consultations, investigations, and prescriptions from nine GP practices in the Keele GP Research Partnership, North Staffordshire, UK.^10,11^ It includes approximately 124 000 registered patients. As the final data entry for the database was in 2016, patients were included till the end of December 2014 to give at least a 1-year follow-up.

Patients were included if they were aged ≥50 years in the study period with Read codes (Supplementary Information S1 and S2) for falls (U10..) or previous fractures in the hip, spine, humerus, or forearm or if they had been prescribed 420 mg prednisolone or more in a 3-month period. Oral steroid use for this study was defined as equivalent to 5 mg or more of prednisolone daily for 3 months (84 days) or more. Read codes form a coding system that aids transfer of patient clinical information between practices and for monitoring healthcare delivery as well as for research purposes. As fragility fractures are unlikely to be coded in the CiPCA database, an assumption was made that a previous fracture at one of the above four sites commonly affected by osteoporotic fractures in a patient aged ≥50 years was a fragility fracture.^12,13^

Patients were excluded if they were either investigated or treated for fragility fractures with medications including bisphosphonates, raloxifene, denosumab, hormone replacement therapy (HRT), or strontium, in the 2 years preceding the study. Patients on HRT (Supplementary Information S3) without a history of investigation or treatment for fractures were included. Patients with bony metastases, myeloma, and primary bone malignancy at the time when the patient with a risk factor was identified were also excluded as they were likely to develop malignancy-related fractures.^14^ Patients with a cancer diagnosis were included in this study as certain medications, for example anti-androgen therapies used in prostate cancer, can increase fracture risk.

As national guidance does not specify a timeframe to carry out a BHA, 1 year was considered by the authors as an adequate period to capture whether assessments were done on at-risk patients, similar to most Quality and Outcomes Framework (QOF) quality indicators in general practice. BHA performed after 1 year within the study period was recorded, but not classed as an adequate response. In this study, evidence of a BHA was defined by presence of codes for FRAX or QFracture, bone density scan or osteoporosis-related codes, prescription of bone-protection medication including HRT, and referral codes to the osteoporosis clinic or rheumatology services.

Two covariates used in this study were patient comorbidity^15^ and number of consultations a patient had in 1 year.^16^ Patient comorbidity was estimated by looking at the drug count of patients.^17^ The number of unique *British National Formulary* (*BNF*) chapter codes in a patient’s prescription data were counted within 1 year from the patient’s inclusion date and divided into three categories: 0–4; 5–9; and ≥10.^18^ The number of consultations a patient had in 1 year from presentation of the fracture risk factor was assessed by recording the number of unique dates with consultation-related codes. Terms related to diagnostic and symptom codes, medication reviews, and secondary care specialist investigation codes were included as consultations. The number of consultations a patient had in 1 year were categorised as: 0–3; 4–6; 7–9; and ≥10. The number of prescriptions and consultations were analysed as categorical variables to improve the clinical interpretability of the model estimates derived. Prescription data cutoff points have been successfully used in previous research.^18^ The cutoff points for the number of consultations were based on clinical knowledge, but also to ensure that there was no one group that contained only a small number of patients. All patients included in this study were used for the final data analysis even if they died within 1 year of inclusion.

### Statistical methods for data analysis

SPSS (version 27) was used for data analysis.^19^ χ^2^ tests were used to assess for statistically strong associations between BHA and patient sex for the sample overall, and stratified by: age (50–74 years versus ≥75 years) and risk factor group (fractures only, falls only, use of steroid only, or ≥2 of the risk factors).

Univariate logistic regression analysis was used to look at the strength of the association between BHA and patient sex. A multivariable logistic regression analysis was then used to adjust for other covariates of interest: age, risk factor group, number of consultations, and patient comorbidity. Age was included as a continuous variable and all other variables were included as categorical variables.

The covariates were all placed in the same model in the multivariable analysis. Results will be presented as odds ratios with 95% confidence intervals.

## Results

**Figure 1.**
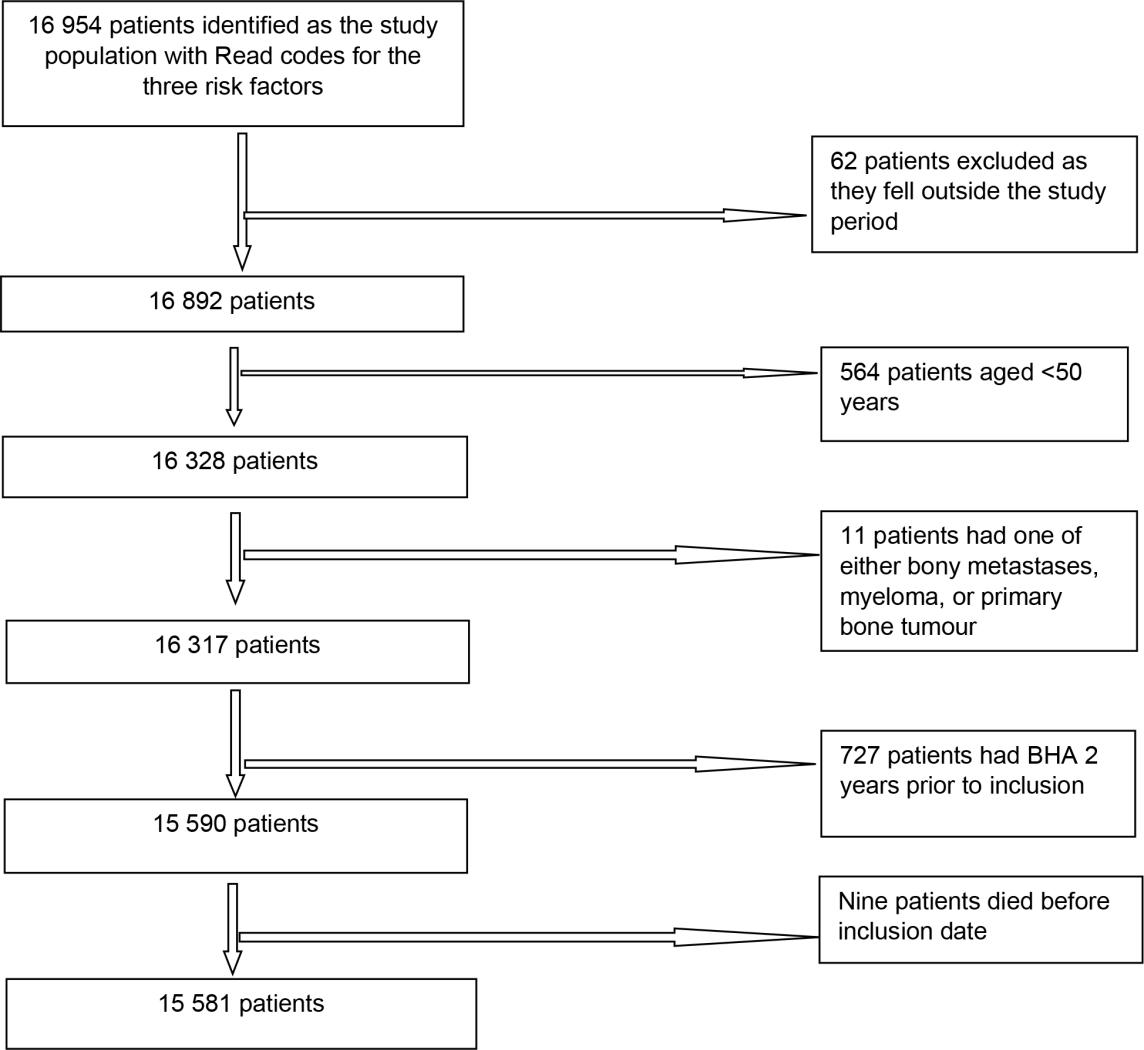
Flowchart of patient identification. BHA = bone health assessment

A total of 16 954 patients were eligible for study inclusion. The number of patients excluded owing to various reasons was 1373, leaving 15 581 patients in the cohort (Figure 1). The mean age was 73.4 years (standard deviation 10.4 years, range 50–106 years). Table 1 shows the descriptive characteristics of patients included in the study.

**Table 1.** Descriptive characteristics of patients in this study

Demographics		Frequency	Percentage
**Age, years**	50–54	838	5.4%
	55–59	855	5.5%
	60–64	1045	6.7%
	65–69	2787	17.9%
	70–74	2450	15.7%
	≥75	7606	48.8%
**Sex**	Male	6302	40.4%
	Female	9279	59.6%
**Ethnic group**	White British	8626	55.4%
	Mixed	4321	27.7%
	Other White	96	0.6%
	Asian, Asian British	63	0.4%
	Black, African, Black British, Other Black	11	0.07%
	Other Ethnic group	19	0.12%
	Unknown	2445	15.7%

Table 2 illustrates the proportion of patients included with respect to their risk factors as well as further demographics related to the four groups. Of the 2329 patients who had two or more risk factors, 122 patients had all three risk factors. In total, 1172 (7.5%) patients in this cohort had a BHA carried out within 1 year of presentation, while 4960 (31.8%) patients had a BHA after 1 year of inclusion, and 9449 (60.6%) patients had no recorded BHA. Table 3 shows the codes documented for those who had a BHA within 1 year of inclusion (*n* = 1479).

**Table 2.** The distribution of risk factors of patients included in this study

Risk factor	Patients, *n*	Percentage of patients	Mean age, standard deviation(range)	Percentage of women
Falls only	9377	60.2%	75.6, 9.1 (50–102)	56.2%
Fracture only	1198	7.7%	69.9, 12.4 (50–106)	77.6%
Use of steroids only	2677	17.2%	67.0, 11.4 (50–102)	55.4%
Two or more risk factors	2329	15.0%	73.7, 9.2 (50–97)	68.7%
Total	15 581	100%	73.4, 10.4 (50–106)	59.6%

**Table 3.** Type of BHA completed within 1 year of inclusion in this study

BHA codes	Number of codes (%)	Number of men (%) of total men’s codes	Number of women (%) of total women’s codes
FRAX or QFracture	8 (0.5%)	4 (0.9%)	4 (0.4%)
Computerised bone densitometry, DXA, or osteoporosis-related codes	842 (56.9%)	231 (53.0%)	611 (58.6%)
Referral to specialist	162 (11%)	64 (14.7%)	98 (9.4%)
HRT prescriptions	20 (1.4%)	Not applicable	20 (1.9%)
Bisphosphonates and other bone protection prescriptions and related codes (bisphosphonates contraindicated, declined, or not indicated)	441 + (6) (30.2%)	135 + (2) (31.4%)	306 + (4) (29.7%)
Total codes	1479 (100%)	436	1043

BHA = bone health assessment. DXA = dual X-ray absorptiometry. HRT = hormone replacement therapy.

Women were found to be more likely to have a BHA compared with men, with 5.5% (*n* = 349) of men and 8.9% (*n* = 823) of women having had a BHA within 1 year of presentation with a fragility fracture risk factor (χ^2^ = 59.88, *P* = 1 x 10^–14^). Table 4 shows differences in BHA rates, by sex stratified by the two age groups (50–74 years, ≥75 years) and risk factor, the relation between BHA with patient sex was present in both age categories (*P* = 1 x 10^–5^, *P* = 6 × 10^–11^, respectively). With respect to the four risk factors; the falls-only group showed a statistically strong difference between the two sexes in relation to BHA (χ*^2^* = 21.86, *P* = 3 × 10^–6^).

**Table 4.** Showing χ^2^ test done to assess difference with patient sex overall and with stratification of age categories and specific risk factors

	Observed (%) having a BHA	Expected (%) having a BHA	χ^2^ (*P* value)
**Overall**			
Male	349 (5.5%)	474 (7.5%)	59.88 (*P* = 1 x 10^–14^)
Female	823 (8.9%)	698 (7.5%)	
**Stratified by age group**			
Age category (**50–74 years**) (*n* = 7975)			
Male	205 (5.9%)	256.1 (7.4%)	19.45 (*P* = 1 x 10^–5^)
Female	384 (8.5%)	332.9 (7.4%)	
Age category (≥**75 years**) (*n* = 7606)			
Male	144 (5.1%)	217.3 (7.7%)	42.69 (*P* = 6 x 10^–11^)
Female	439 (9.2%)	365.7 (7.7%)	
**Stratified by risk factor**			
**Fractures** (*n* = 1198)			
Male	75 (28%)	72.5 (27.1%)	0.16 (*P* = 0.69)
Female	249 (26.8%)	251.5 (27%)	
**Falls** (*n* = 9377)			
Male	52 (1.3%)	83.7 (2%)	21.86 (*P* = 3 × 10^–6^)
Female	139 (2.6%)	107.3 (2%)	
**Steroids** (*n* = 2677)			
Male	124 (10.4%)	139.3 (11.7%)	3.43 (*P* = 0.06)
Female	188 (12.7%)	172.7 (11.7%)	
**Two or more risk factors** (*n* = 2329)			
Male	98 (13.5%)	107.8 (14.8%)	1.53 (*P* = 0.22)
Female	247 (15.4%)	237.2 (14.8%)	

Observed = actual results. Expected = number of patients who would fall in each category if no association between the variables exists. BHA = bone health assessment.

Table 5 outlines the univariate followed by multivariate regression analysis of BHA. On univariate analysis, the odds ratio of women having a BHA compared with men was found to be 1.66 (95% CI = 1.46 to 1.89). Following adjustment for other variables (age, patient comorbidity, number of consultations, and nature of risk factor) the odds ratio for BHA for women compared with men remained statistically strong at 1.25 (95% CI = 1.08 to 1.43; *P* = 0.002).

**Table 5.** Univariate analysis of patient sex on BHA followed by multivariate analysis of various factors affecting BHA including, patient age, comorbidity, number of consultations, and type of risk factor

Risk factor	Odds ratio for BHA	95% confidence interval	*P* value
Female sex (univariate analysis)	1.66	1.46 to 1.89	2 × 10^–14^
Female sex (multivariate analysis)	1.25	1.08 to 1.43	0.002
Age	1.03	1.02 to 1.03	1 × 10^–15^
Consultation category (0–3), ref			
Consultation category (4–6)	3.20	2.00 to 5.10	1 × 10^–6^
Consultation category (7–9)	4.36	2.76 to 6.88	3 × 10^–10^
Consultation category ( ≥10)	8.58	5.57 to 13.22	2 × 10^–22^
Comorbidity (No. *BNF* chapters 0–4), ref			
Comorbidity (No. *BNF* chapters 5–9)	0.97	0.64 to 1.45	0.864
Comorbidity (No. *BNF* chapters ≥10)	0.64	0.43 to 0.96	0.032
Risk factor fall, ref			
Fracture	21.01	17.16 to 25.71	2 × 10^–191^
Steroid use	7.16	5.89 to 8.72	4 × 10^–86^
Two or more risk factors	8.22	6.80 to 9.95	8 × 10^–104^

BHA = bone health assessment. *BNF* = *British National Formulary.* ref = reference.

Of the 940 patients in this cohort who died before their 1-year follow-up from the inclusion date, 58 patients had a BHA before their death.

## Discussion

### Summary

This retrospective cohort study in North Staffordshire, UK, has demonstrated that only 5.5% of men and 8.9% of women had a BHA within 1 year of recording of at least one of three major risk factors. After adjustment, women were found to be 25.0% more likely than men to be assessed. The strength of association between patient sex and BHA did not vary with age. However, when stratifying this relation with respect to reason for BHA, falls was the only statistically strong risk factor showing a difference in rates of BHA with patient sex.

### Strengths and limitations

This is the first study to the authors' knowledge to examine sex differences with respect to BHA in patients from a UK primary care population who are at risk of osteoporotic fractures. A strength of this study was the large sample size of nearly 16 000 patients (60% women and 40% men). Compared with other published articles, this study is unique as it includes three risk factors including fractures originating from four sites: hip, spine, humerus, and forearm. Several authors have only looked at patients with previous hip fractures,^20–22^ while other studies^9,23^ have reviewed patients from certain age groups. Incident patients were only included in this study, so the risk of bias from previous investigations and treatments was minimised.

In this study, BHA determination was based on clinical coding. Relying on coding may underestimate BHA, as text in some GP consultations may not have been coded, possibly underestimating rates of BHA. FRAX risk assessment was less frequently recorded than DXA and osteoporosis medicines and it is possible this is less likely to be recorded; this could potentially lead to bias in the results as men are likely to be lower-fracture risk and less likely to need onward referral for DXA and/or medicine. A further limitation of this study relates to the study period being from 2002–2014, while National Institute for Health and Care Excellence (NICE) guidance (CG146)^3^ on osteoporosis was first published in 2012 and reupdated in 2017.^24^ Recent evidence from a single centre in Italy has suggested that sex differences in BHA persist.^25^ Although more contemporaneous data from the UK are needed, this study represents the best available evidence on this issue.

As only patients with prednisolone use were included, this study cannot assess current BHA practice in patients on other oral steroids such as dexamethasone. However, data from a General Practice Research Database (GPRD)-based study^26^ has shown prednisolone accounted for more than 90% of oral steroid prescriptions, suggesting this study’s results are relevant to most patients on oral steroids. The study also did not examine the rate of falls risk assessment, which is a further important assessment when considering fracture risk, particularly in those with prior fractures. A further limitation is that the data are generated from North Staffordshire, the population of which may not be representative of the wider UK population.

### Comparison with existing literature

This study’s finding of men having a lower rate of BHA compared with women concurs with other published articles.^9,27^ Codes for FRAX and QFracture were used in only 0.5% of the total BHA codes (Table 3), which could be an underestimate. In a Canadian multicentre osteoporosis study looking at men aged ≥50 years, only 2.3% of men with fragility fractures in the cohort were diagnosed with osteoporosis at the start of the study, which increased to 10.3% at 5-year follow-up.^28^ This suggests healthcare providers do not seem to relate fragility fractures in men to the diagnosis of osteoporosis. Underdiagnosis of men with osteoporosis does seem to translate to reduced treatment rates.^29–33^ Curtis *et al*’s study,^23^ in more than 24 000 patients aged ≥45 years, demonstrated that the odds ratio of men receiving osteoporosis treatment compared with women was 0.08 (95% CI = 0.06 to 0.10).

### Implications for research and practice

This study’s findings demonstrate low BHA rates (7.5%) in both women and men with men statistically less likely to have a BHA compared with women. A reduced rate of BHA in both men and women means that opportunities for fracture prevention are lost, potentially leading to fractures that could have been avoided. This study has shown a very low recording or coding of FRAX and QFracture in primary care (0.5%). Identifying high-risk patients with an improved use of these tools could potentially improve clinical management of these patients. A randomised controlled trial,^34^ carried out in multiple GP practices in the UK, demonstrated that FRAX-based screening in women aged 70–85 years improved rates of osteoporosis treatment compared with standard care (15% versus 4%) and lowered rates of hip fractures.

Previous qualitative studies have identified barriers to identification and management of osteoporosis in primary care, including perceptions that osteoporosis is low priority, ambivalence about the safety and effectiveness of medication, and uncertainty about clinical guidelines.^35,36^

In addition to addressing these barriers, patient campaigns are needed to increase awareness of this clinical problem,^37^ and primary care incentivisation may help to improve implementation of fracture risk assessments.^38^

Further research in more contemporaneous datasets (reporting data from 2014 to present) is needed to establish if BHA rates remain low, and if men remain underrepresented. Furthermore, research is needed to identify other possible characteristics associated with reduced likelihood of receiving BHAs in deprived and underserved communities to enable further strategies to target specific high-risk groups.

In conclusion, this study provides evidence that low rates of BHA were carried out in patients with risk factors especially in men. Osteoporosis is a ‘silent disease’, which has far-reaching social, economic, and clinical consequences. Reducing the burden of osteoporosis starts with primary prevention and primary care clinicians are advised to be alert for fragility fracture risk factors in both men and women, and proactively engage in early assessment and intervention where appropriate. This may be promoted by financial incentives, support from healthcare commissioners in providing access to relevant services, and policymakers when developing national health strategy.

## Data

Data from the CiPCA database is not available for publication due to patient confidentiality.
